# Effect of Genetic Variability in the *CYP4F2*, *CYP4F11*, and *CYP4F12* Genes on Liver mRNA Levels and Warfarin Response

**DOI:** 10.3389/fphar.2017.00323

**Published:** 2017-05-31

**Authors:** J. E. Zhang, Kathrin Klein, Andrea L. Jorgensen, Ben Francis, Ana Alfirevic, Stephane Bourgeois, Panagiotis Deloukas, Ulrich M. Zanger, Munir Pirmohamed

**Affiliations:** ^1^Wolfson Centre for Personalized Medicine, Department of Molecular and Clinical Pharmacology, The University of LiverpoolLiverpool, United Kingdom; ^2^Dr. Margarete Fischer-Bosch Institute of Clinical PharmacologyStuttgart, Germany; ^3^Department of Clinical Pharmacology, University of TuebingenTuebingen, Germany; ^4^Department of Biostatistics, The University of LiverpoolLiverpool, United Kingdom; ^5^William Harvey Research Institute, Barts and the London School of Medicine and Dentistry, Queen Mary University of LondonLondon, United Kingdom; ^6^Wellcome Trust Sanger InstituteCambridge, United Kingdom; ^7^Princess Al-Jawhara Al-Brahim Centre of Excellence in Research of Hereditary Disorders, King Abdulaziz UniversityJeddah, Saudi Arabia

**Keywords:** warfarin, pharmacogenetics, mRNA expression, CYP4F2, CYP4F11, CYP4F12

## Abstract

Genetic polymorphisms in the gene encoding cytochrome P450 (CYP) 4F2, a vitamin K oxidase, affect stable warfarin dose requirements and time to therapeutic INR. *CYP4F2* is part of the *CYP4F* gene cluster, which is highly polymorphic and exhibits a high degree of linkage disequilibrium, making it difficult to define causal variants. Our objective was to examine the effect of genetic variability in the *CYP4F* gene cluster on expression of the individual *CYP4F* genes and warfarin response. mRNA levels of the *CYP4F* gene cluster were quantified in human liver samples (*n* = 149) obtained from a well-characterized liver bank and fine mapping of the *CYP4F* gene cluster encompassing *CYP4F2*, *CYP4F11*, and *CYP4F12* was performed. Genome-wide association study (GWAS) data from a prospective cohort of warfarin-treated patients (*n* = 711) was also analyzed for genetic variations across the *CYP4F* gene cluster. In addition, SNP-gene expression in human liver tissues and interactions between *CYP4F* genes were explored *in silico* using publicly available data repositories. We found that SNPs in *CYP4F2*, *CYP4F11*, and *CYP4F12* were associated with mRNA expression in the *CYP4F* gene cluster. In particular, *CYP4F2* rs2108622 was associated with increased *CYP4F2* expression while *CYP4F11* rs1060467 was associated with decreased *CYP4F2* expression. Interestingly, these *CYP4F2* and *CYP4F11* SNPs showed similar effects with warfarin stable dose where *CYP4F11* rs1060467 was associated with a reduction in daily warfarin dose requirement (∼1 mg/day, *P_c_* = 0.017), an effect opposite to that previously reported with *CYP4F2* (rs2108622). However, inclusion of either or both of these SNPs in a pharmacogenetic algorithm consisting of age, body mass index (BMI), gender, baseline clotting factor II level, *CYP2C9^∗^2* rs1799853, *CYP2C9^∗^3* rs1057910, and *VKORC1* rs9923231 improved warfarin dose variability only by 0.5–0.7% with an improvement in dose prediction accuracy of ∼1–2%. Although there is complex regulation across the *CYP4F* gene cluster, the opposing effects between the two SNPs in the *CYP4F* gene cluster appear to compensate for each other and their effect on warfarin dose requirement is unlikely to be clinically significant.

## Introduction

The *CYP4F* gene subfamily comprises six members, namely *CYP4F2* (Kikuta et al., 1993), *CYP4F3* (*CYP4F3A* and *CYP4F3B*) (Kikuta et al., 1998), *CYP4F8* (Bylund et al., 2000), *CYP4F11* (Cui et al., 2000), *CYP4F12* (Bylund et al., 2001; Hashizume et al., 2001), and *CYP4F22* (Lefevre et al., 2006). Structurally, these six *CYP4F* genes are largely similar, with more than 65% amino acid sequence homology. To date, studies have focused on *CYP4F2, CYP4F3, CYP4F8, CYP4F11*, and *CYP4F12* while little is known about the expression and function of *CYP4F22*. The splice sites of *CYP4F2, CYP4F3, CYP4F8, CYP4F11*, and *CYP4F12* are almost identical, suggesting that this cluster of five genes may have evolved by gene duplication (Bylund et al., 1999, 2001; Kikuta et al., 1999; Cui et al., 2000).

*CYP4F2, CYP4F3, CYP4F8, CYP4F11*, and *CYP4F12* reside together on chromosome 19p13.1-2, spanning over 320 kb (Supplementary Figure 1). These five members of the *CYP4F* subfamily are all expressed in the liver and are known for their roles in the metabolism of both endogenous and exogenous compounds. They are involved in the catabolism of substrates such as arachidonic acid and its oxygenated derivatives (eicosanoids) such as leukotrienes, prostaglandins (PGs), lipoxins, and hydroxyeicosatetraenoic acids (HETEs) (Kikuta et al., 1999; Bylund et al., 2000, 2001; Hashizume et al., 2001, 2002; Kalsotra et al., 2004), and they also catalyze the metabolism of many drugs. For example, *CYP4F2* has also been implicated in the ω-hydroxylation of the tocopherol phytyl side chain in the first step of vitamin E inactivation (Sontag and Parker, 2002). In addition, *CYP4F2* and *CYP4F3B* have been shown to catalyze the initial *O*-demethylation of the anti-parasitic prodrug pafuramidine by human liver and intestinal microsomes (Wang et al., 2006, 2007). *CYP4F2* has also been reported to be a vitamin K oxidase and plays a role in warfarin response (McDonald et al., 2009). *CYP4F11* is known to be active in the metabolism of several drugs including erythromycin, benzphetamine, ethylmorphine, chlorpromazine, and imipramine (Kalsotra et al., 2004). More recently, a study has reported that *CYP4F11* functions as a vitamin K ω-hydroxylase (Edson et al., 2013). *CYP4F12* has been reported to be involved in the conversion of the antihistaminic prodrug ebastine to the active drug carebastine by hydroxylation (Hashizume et al., 2001, 2002).

Warfarin is one of the most widely used oral anticoagulants worldwide with proven efficacy in conditions characterized by thromboembolism including atrial fibrillation, deep vein thrombosis, pulmonary embolism, or heart valve prostheses. Despite its efficacy, warfarin is often among the top three drugs that lead to hospitalization from adverse drug reactions (Budnitz et al., 2007; Wysowski et al., 2007), owing to its narrow therapeutic window and large inter-individual variability in dose response. Combinations of both non-genetic and genetic factors influence the inter-individual variability in warfarin therapeutic dose requirements. Genetic factors, in particular, single nucleotide polymorphisms (SNPs) in two genes responsible for warfarin pharmacokinetics and pharmacodynamics – cytochrome P450 2C9 (*CYP2C9*) and vitamin K epoxide reductase complex 1 (*VKORC1*) – have repeatedly been found to be significantly associated with warfarin responsiveness, explaining approximately 15 and 25% of dose variability (Aithal et al., 1999; Yuan et al., 2005; Gage et al., 2008; Wadelius et al., 2009; Schwanhausser et al., 2011), respectively. Candidate gene(s) and GWAS studies have shown that the *CYP4F2* functional variant, rs2108622, accounts for a small proportion of the variability in warfarin dose requirement (1–7%) (Caldwell et al., 2008; Borgiani et al., 2009; Takeuchi et al., 2009; Pautas et al., 2010). However, some studies have not found an association between rs2108622 and warfarin stable dose (Zhang et al., 2009; Perini et al., 2010). A functional study utilizing human liver tissues did not find any association between rs2108622 and *CYP4F2* mRNA but observed a significant association between the rs2108622 variant TT genotype and lower microsomal CYP4F2 protein concentration and reduced vitamin K_1_ oxidation, consistent with its function as a vitamin K_1_ oxidase in catalyzing the ω-hydroxylation of vitamin K_1_ phytyl side chain (McDonald et al., 2009).

We have previously performed fine mapping of the *CYP4F2* region to determine the influence of *CYP4F2* SNPs and haplotypes on various warfarin response outcomes (Zhang et al., 2009). We found an association between rs2189784, a SNP in strong linkage disequilibrium (LD) with rs2108622, with time to achieve therapeutic International Normalized Ratio (INR), but not with stable dose. Given the high degree of homology and LD across the *CYP4F* gene cluster (Supplementary Figure 1), we have undertaken a genotype–phenotype assessment utilizing a well-characterized liver bank and a prospective patient cohort who were followed up for 6 months from the time of intake of warfarin (as summarized in Supplementary Figure 2). *In silico* analysis was also performed to investigate additional SNP-gene associations and the interactions between the *CYP4F* genes.

## Materials and Methods

### Study Populations

Written informed consent in accordance with the Declaration of Helsinki was obtained from all patients recruited to the following cohorts.

#### Liver Surgery Cohort

Blood and liver tissue samples were collected from 149 Caucasian patients undergoing liver surgery at the Department of General, Visceral, and Transplantation Surgery, Campus Virchow, University Medical Centre Charité, Humboldt University, Berlin, Germany, as described previously (Gomes et al., 2009). Normal liver tissues were obtained from adjacent regions of surgically removed liver tumors or metastases or hepatic tissue respected for other reasons. All liver tissue samples were certified to be free of malignant cells by pathological examination. None of these samples were from patients with hepatitis, or cirrhosis, or from those who had chronic alcohol abuse. Clinical patient documentation for all samples included age, gender, medical diagnosis, pre-surgical medication, alcohol use, and smoking. The study was approved by the Research Ethics Committees of the Medical Faculties of the Charité, Humboldt University, Berlin, and of the University of Tuebingen, Tuebingen, Germany.

#### Warfarin-Treated Patient Cohort

Thousand patients starting warfarin therapy were recruited prospectively at two hospitals in Liverpool, United Kingdom (Royal Liverpool and Broadgreen University Hospitals Trust and University Hospital Aintree). The main indications for warfarin therapy were treatment of venous thromboembolism and prophylaxis against systemic emboli in patients with atrial fibrillation. The study was approved by the Birmingham South Research Ethics Committee, United Kingdom.

### Determination of *CYP4F2, CYP4F8, CYP4F11*, and *CYP4F12* mRNA Expression Levels in Human Liver

RNA was extracted from the human liver tissue (*n* = 149) using TRIzol^®^ reagent (Invitrogen, Paisley, United Kingdom) with subsequent RNA clean-up using QIAGEN RNeasy-Mini Kit with on-column DNase treatment. All RNA preparations were of high quality with RNA integrity number (RIN) >7, as measured on the Agilent Bioanalyzer (Nano-Lab Chip Kit, Agilent Technologies, Waldbronn, Germany). Levels of gene expression of over 48,000 mRNA transcripts were assessed by the Human-WG6v2 Expression BeadChip (Illumina, Eindhoven, The Netherlands) as previously described (Schroder et al., 2013). Pre-processing and quality control of the expression data was conducted using the Illumina BeadStudio, version 3.0 (Illumina, San Diego, CA, United States) and the various steps involved are detailed in Schroder et al. (2013). Probe signal intensities corresponding to 15,439 unique genes remain after all pre-processing steps and the data set was log2 transformed. Probe sequences for *CYP4F2*, *CYP4F8*, *CYP4F11*, and *CYP4F12* were confirmed to be specific and expression data were extracted. *CYP4F3* and *CYP4F22* were not further analyzed due to ambiguous probe or gene annotation.

#### Liver Surgery Cohort: *CYP4F2, CYP4F11, CYP4F12* SNP Selection, Genotyping, and Haplotype Analysis

Genomic DNA from the liver surgery patients (*n* = 149) was extracted from whole blood using the QIAamp DNA Mini Kit (QIAGEN GmbH, Hilden, Germany) according to the manufacturer’s instructions.

Eighty genetic polymorphisms in the *CYP4F2* gene were selected as previously reported (Zhang et al., 2009). SNPs encompassing *CYP4F11* and *CYP4F12* across the chromosomal 19p13.11 region were chosen on the basis of their functionality, coverage in the CEU population (Utah residents with ancestry from northern and western Europe) available on HapMap data release 27, NCBI build 36 assembly, minor allele frequency (MAF > 1%) and block-tagging ability (*r*^2^ ≥ 0.8). A total of 130 SNPs in the *CYP4F11* and *CYP4F12* region were successfully designed and subdivided into six multiplex assays using Sequenom’s online Human GenoTyping Tools^1^. Primer sequences are available on request. All SNPs were genotyped using the Sequenom MassARRAY iPLEX^TM^ platform (Sequenom, Hamburg, Germany) in accordance with the manufacturer’s instructions. To ensure data quality, 10% DNA replicates and 8 negative controls (water) were included per 384-well plate during genotyping. Markers which deviated from Hardy–Weinberg equilibrium (HWE, *P* < 0.001) (*n* = 10), those with less than 90% call rate (*n* = 27), and those which were monomorphic (*n* = 26), were excluded from downstream analysis (see Supplementary Table 1).

The pattern of pairwise LD between the SNPs was visualized using the program HaploView version 4.2 (Barrett et al., 2005). Haplotype blocks were defined using the default algorithm by Gabriel et al. (2002) in HaploView. The most probable combinations of haplotype-pairs at each block were inferred using the program PHASE version 2.1.1 (Stephens et al., 2001; Stephens and Scheet, 2005). Any individuals with a haplotype-pair probability of <90% (*n* = 11) for at least one haplotype block were excluded from tests of association. Within a haplotype block, haplotypes with frequencies <1% were grouped together as a single covariate for analysis.

#### Warfarin-Treated Patient Cohort: Genome-Wide Genotyping and Imputation

Genomic DNA was extracted from whole blood using the standard phenol–chloroform method. Genome-wide genotyping was performed using the Illumina Human610-Quad BeadChip (Illumina, San Diego, CA, United States) at the Wellcome Trust Sanger Institute, United Kingdom. Of the 1000 patients recruited, genome-wide genotype data were available for 752 individuals as previously described (Bourgeois et al., 2016). All quality control measures were performed using PLINK (Purcell et al., 2007). All SNPs with a genotyping success rate <95%, HWE threshold of *P* < 0.0001 and those with MAF <1% were excluded from the dataset. Cryptic relatedness was assessed between individuals and one individual from each pair with an estimated identity by descent (IBD) >0.1875 (i.e., halfway between the expected IBD for third- and second-degree relatives) was removed. Subjects with genotyping success rate <95% were also removed. Principle component analysis was performed to assess genetic markers for ethnicity. Only individuals with genetically matching ethnicity were included into the association analysis (*n* = 711).

For the purpose of this study, genotype data for SNPs across *CYP4F2*, *CYP4F11*, and *CYP4F12* were extracted (*n* = 80) for downstream analyses. After pre-phasing via SHAPEIT (Delaneau et al., 2012), imputation of genotypes at additional SNPs throughout the region encompassing *CYP4F2*, *CYP4F11*, and *CYP4F12* (chr19:15–17 Mb, B36) was carried out using IMPUTE2 (Howie et al., 2009) with the reference genotype data from the European 1000 Genomes Phase I data set (release date June 2014). Imputed variants with an information score <0.8, MAF <1%, HWE <0.0001 and genotyping success rate <95% were excluded using QCTool, leaving a total of 1400 SNPs harboring the *CYP4F12-CYP4F2-CYP4F11* genomic region.

### Statistical Analysis

Statistical analyses were conducted with the software package SPSS, version 18. For each univariate test of association, two tests were performed, one making no assumption on the mode of inheritance while the other assumed an additive mode of inheritance. The minimum *P*-value is referred to in each analysis.

All *P*-values from the genotype–phenotype association tests undertaken in the functional and clinical studies were independently adjusted for multiple testing using false discovery rate (FDR) (Benjamini et al., 2001) in the genetics package of R, version 3.1.2^2^. FDR-corrected *P*-values are denoted as *P_c_*-values and values <0.05 were regarded as statistically significant.

The proportion of variability explained by the genetic covariates was calculated using Nagelkerke’s *R*^2^ statistic (Nagelkerke, 1991).

#### Liver mRNA Analysis

Relationships between each of the phenotypic parameters evaluated were examined by Spearman correlation analysis. The mRNA levels of the four *CYP4F* genes were not normally distributed. To enable the use of parametric statistical tests, the expression data were natural log transformed. To evaluate the association of each SNP or haplotype with mRNA expression levels, one-way analysis of variance (ANOVA) and univariate linear regression were conducted.

#### Warfarin Outcome Analysis

Warfarin stable dose was defined as an unchanged daily dose at three or more consecutive clinic visits where INR measurements were within the individual’s target range (Jorgensen et al., 2009; Zhang et al., 2009). As the distribution of stable dose was skewed, the outcome was log transformed to achieve normal distribution. To test for the association of SNPs with warfarin stable dose, ANOVA and univariate linear regression were employed. Conditional analysis was conducted by including the SNP of interest into the linear regression model as a covariate. Dosing algorithms were built by incorporating significant (*P* ≤ 0.05) clinical and genetic variables from the univariate analyses into the multiple linear regression models. Supplementary Table 3 reports the significant results of the univariate analyses. To assess the predictive accuracy of the dosing algorithms, the mean absolute error was determined by calculating the average of the difference between the predicted and actual stable doses. The percentage of predicted dose which fell within 20% of the actual maintenance dose was also calculated.

### *In Silico* Analysis to Identify SNPs Associated with mRNA Expression

Putative expression quantitative trait loci (eQTLs) in the *CYP4F* gene cluster were identified using the eQTL browser^3^, a database that summarizes results from large-scale studies which identified eQTLs in the liver (Schadt et al., 2008), brain (Myers et al., 2007), fibroblasts (Dimas et al., 2009), T-cells (Dimas et al., 2009), monocytes (Zeller et al., 2010), and lymphoblastoid cell lines (Stranger et al., 2007; Veyrieras et al., 2008; Dimas et al., 2009; Montgomery et al., 2010; Pickrell et al., 2010).

### Network Building to Evaluate Interaction between CYP4F2, CYP4F11, and CYP4F12

The interactions between CYP4F2, CYP4F11, and CYP4F12 were visualized in MetaCore^TM^ (GeneGo, Inc., St. Joseph, MI, United States), an interactive database derived from manually curated literature publications on proteins and small molecules of biological relevance in humans. Results are discussed in the Supplementary Material (Supplementary Figure 3, Supplementary Results, and Supplementary Discussion).

## Results

### Correlation between Hepatic *CYP4F* mRNA Expression Levels

**FIGURE 1:**
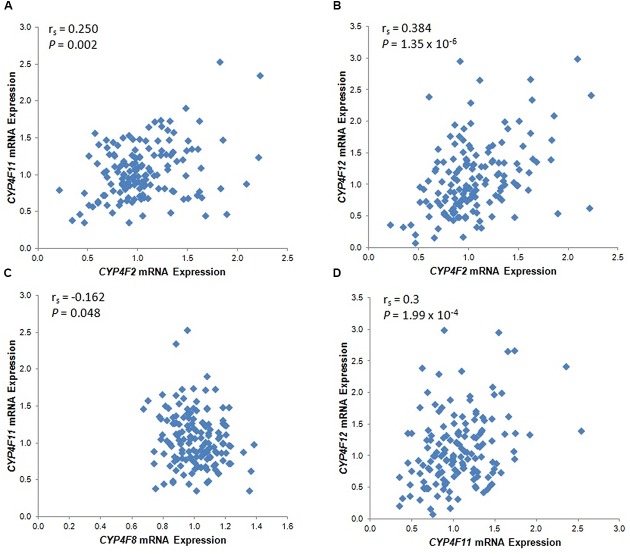
Statistically significant *CYP4F* mRNA correlations in 149 Caucasian human liver tissues. **(A)** *CYP4F2* vs. *CYP4F11*; **(B)** *CYP4F2* vs. *CYP4F12*; **(C)** *CYP4F8* vs. *CYP4F11;* **(D)** *CYP4F11* vs. *CYP4F12*. vs., versus. The Spearman’s rho correlation coefficient (r_s_) and *P*-value for each comparison are given. Log2 transformed expression data are presented.

The mRNA levels of the four *CYP4F* genes that were detected by specific probes varied considerably between individuals, ranging from an expression ratio of 2 for *CYP4F8* to an expression ratio of 37 for *CYP4F12* (**Table 1**). Significant correlations among the four *CYP4F* genes are depicted in **Figure 1**. *CYP4F11* and *CYP4F12* mRNA showed significant albeit not very strong correlations with *CYP4F2* mRNA (*r*_s_ = 0.25 and 0.384, respectively, *P* < 0.01; **Figures 1A,B**) and with each other (*r*_s_ = 0.3, *P* < 0.001; **Figure 1D**). *CYP4F8* expression was not significantly correlated to any of the others.

**Table 1:** Variability of mRNA expression in the *CYP4F* gene cluster.

Gene	*n*	mRNA expression (arbitrary unit)	
		Range	Ratio (maximum/minimum)
*CYP4F2*	149	0.21–2.22	10
*CYP4F8*	149	0.67–1.38	2
*CYP4F11*	149	0.35–2.53	7
*CYP4F12*	149	0.08–2.99	37

*Normalized median mRNA expression for all genes = 1.00*.

### Genotype–Phenotype Correlation between *CYP4F2* Variants and Hepatic mRNA Expression of the *CYP4F* Gene Cluster

**FIGURE 2:**
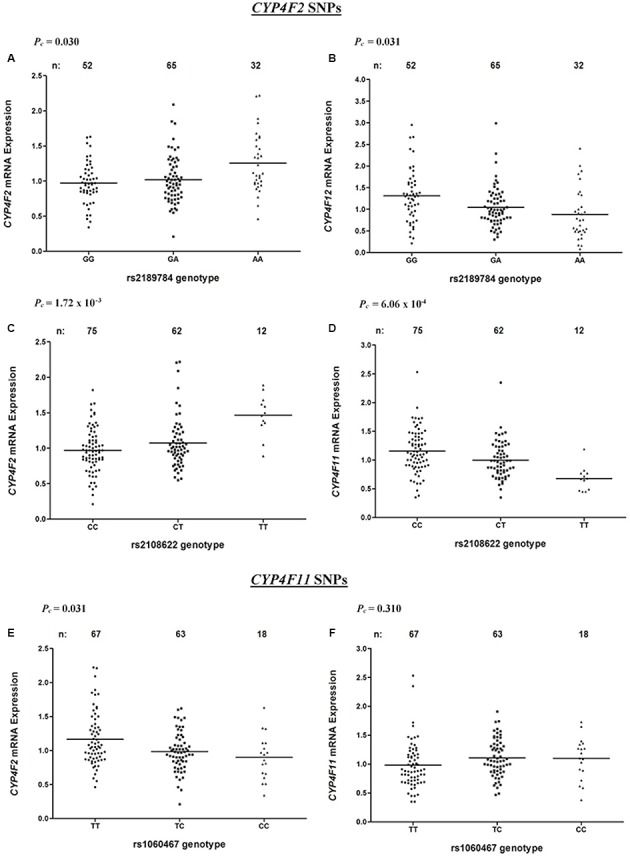
Levels of *CYP4F2*, *CYP4F11*, *CYP4F12* mRNA in normal liver tissue donated from 149 patients in relation to corresponding SNPs across the *CYP4F2*-*CYP4F11* locus. **(A,B)** rs2189784; **(C,D)** rs2108622; **(E,F)** rs1060467. *FDR*-corrected *P*-values (*P_c_*) are shown in the upper left corner. Each dot represents an individual and the solid lines represent the mean values.

Associations between *CYP4F2* variants and hepatic mRNA expression of the *CYP4F* gene cluster are summarized in **Table 2**. Contrary to a previous report (McDonald et al., 2009), we found a significant association between rs2108622 and liver *CYP4F2* mRNA expression (**Figure 2C**), with subjects homozygous for the rs2108622 minor T allele showing greater *CYP4F2* expression compared to subjects homozygous for the major C allele (TT = 1.47 ± 0.29, CC = 0.97 ± 0.31, *P_c_* = 1.72 × 10^-3^, *R*^2^ = 12.6%). Moreover, several other *CYP4F2* variants were also associated with significantly higher *CYP4F2* expression including rs2189784 (*P_c_* = 0.030, *R*^2^ = 7.9%, **Figure 2A**), a SNP located 29 kb downstream of the *CYP4F2* gene.

**Table 2:** Genotype–phenotype correlation of *CYP4F2, CYP4F11*, and *CYP4F12* SNPs and hepatic mRNA expression.

			mRNA expression								
			*CYP4F2*			*CYP4F11*			*CYP4F12*		
Gene	SNP	Localization	*P*-value	↑ or ↓	*R*^2^ (%)	*P*-value	↑ or ↓	*R*^2^ (%)	*P*-value	↑ or ↓	*R*^2^ (%)
**SNPs in *CYP4F2* region**											
*–*	rs2189784	Downstream of *CYP4F2*	**0.030**	**↑**	**7.9**	0.315	–	–	**0.031**	↓	**8.3**
*CYP4F2*	rs3093209^∗^	3′ near gene	**1.68 × 10^-3^**	**↑**	**14.6**	0.097	–	–	0.276	–	–
*CYP4F2*	rs2108622	Exon 11, Missense, Met433Val	**1.72 × 10^-3^**	**↑**	**12.6**	**6.06 × 10^-4^**	**↓**	**13.7**	0.509	–	–
*CYP4F2*	rs3093173^∗^	Intron 9	0.485	–	–	**0.034**	**↓**	**7.2**	0.481	–	–
*CYP4F2*	rs3093169^∗^	Intron 9	0.655	–	–	**0.035**	**↓**	**5.8**	0.275	–	–
*CYP4F2*	rs984692	Intron 3	0.519	–	–	0.512	–	–	0.125	–	–
**SNPs in *CYP4F11* and *CYP4F12***											
*CYP4F11*	rs1060467	3′ UTR	**0.031**	**↓**	**7.2**	0.310	–	–	0.632	–	–
*CYP4F11*	rs12977516^∗^	Intron 8	0.065	–	–	**0.043**	**↑**	**6.4**	0.874	–	–
*CYP4F11*	rs1471112	Intron 8	0.830	–	–	0.867	–	–	0.868	–	–
*CYP4F11*	rs11086012	Intron 8	0.891	–	–	0.715	–	–	0.650	–	–
*CYP4F11*	rs7249167	Intron 4	0.925	–	–	0.479	–	–	0.407	–	–
*CYP4F12*	rs17682485	Intron 3	0.455	–	–	0.989	–	–	**0.034**	**↓**	**7.9**
*CYP4F12*	rs12460703	Intron 3	0.478	–	–	0.998	–	–	**0.016**	**↑**	**8.9**
*CYP4F12*	rs2074568	Intron 4	0.920	–	–	0.429	–	–	**5.43 × 10^-5^**	**↑**	**19.5**
*CYP4F12*	rs10409750	Intron 5	0.692	–	–	0.844	–	–	**3.25 × 10^-4^**	**↓**	**14.8**
*CYP4F12*	rs10410357	Intron 9	0.761	–	–	0.915	–	–	**6.09 × 10^-3^**	**↓**	**10.3**
*CYP4F12*	rs11879787	Intron 9	0.941	–	–	0.805	–	–	**3.22 × 10^-5^**	**↑**	**17.9**
*CYP4F12*	rs627971^∗^	Intron 9	0.497	–	–	0.912	–	–	**0.032**	**↓**	**7.1**
*CYP4F12*	rs2886476^∗^	3′ near gene	0.869	–	–	0.861	–	–	**3.84 × 10^-5^**	**↑**	**18.4**

*Statistically significant results with a P < 0.05 are shown in bold. R^2^ refers to coefficient of determination. ^∗^Tagging SNP (r^2^ ≥ 0.9). Tagged SNPs are detailed in Supplementary Table 2.*

Interestingly, in addition to being associated with increased *CYP4F2* mRNA expression, rs2108622 demonstrated a significant association with decreased *CYP4F11* mRNA levels (*P_c_* = 6.06 × 10^-4^, *R*^2^ = 13.7%, **Figure 2D**) while rs2189784 was significantly associated with lower levels of *CYP4F12* mRNA expression (*P_c_* = 0.031, *R*^2^ = 8.3%, **Figure 2B**). No associations were found between *CYP4F2* variants and *CYP4F8* mRNA expression (data not shown).

### Genotype–Phenotype Correlation between *CYP4F11* and *CYP4F12* SNPs and Hepatic mRNA Expression of the *CYP4F* Gene Cluster

Looking at the region encompassing the *CYP4F* gene cluster on HapMap database (Supplementary Figure 1), high LD is seen in the *CYP4F12-CYP4F2-CYP4F11* locus, suggesting that SNPs across the *CYP4F11* and *CYP4F12* regions could be associated with mRNA expression of *CYP4F2* and possibly other *CYP4F* gene cluster members. To examine the genetic contribution of variants in *CYP4F11* and *CYP4F12* on the hepatic mRNA expression of the *CYP4F* gene cluster, fine mapping of the *CYP4F11* and *CYP4F12* gene regions was conducted and significant associations are summarized in **Table 2**.

rs1060467, a genetic variant located in the 3′ untranslated region (UTR) of *CYP4F11* demonstrated a significant association with decreased *CYP4F2* mRNA expression (*P_c_* = 0.031, *R*^2^ = 7.2%, **Figure 2E**); whilst an opposite trend for increasing *CYP4F11* mRNA expression was observed which was not statistically significant after FDR (*P_c_* = 0.310, **Figure 2F**).

Eight SNPs spanning *CYP4F12* were significantly associated with *CYP4F12* mRNA expression. No significant association with *CYP4F8* mRNA expression was observed with any SNPs in the *CYP4F11* or *CYP4F12* region (data not shown).

### Association of Haplotypes in the *CYP4F12-CYP4F2-CYP4F11* Region on Hepatic mRNA Expression of the *CYP4F* Gene Cluster

**FIGURE 3:**
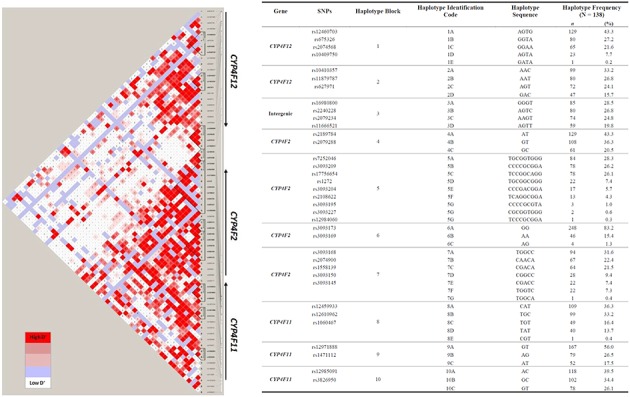
Pairwise LD among polymorphisms in the region of *CYP4F12-CYP4F2-CYP4F11* genes in 149 Caucasian samples (*r*^2^ ≥ 0.9). The left panel shows 10 distinct haplotype blocks defined by the confidence interval algorithm in HaploView 4.2 and the strength of LD is shown in increasing shades of gray, as depicted by the bars. The right panel details the haplotypes sequences and their frequencies inferred by PHASE 2.1.

To explore the complex genetic architecture of *CYP4F* locus containing *CYP4F2*, *CYP4F11* and *CYP4F12*, haplotypes across these three genes were constructed based on the genotype data. Ten haplotype blocks were identified as shown in **Figure 3**, with details of haplotypes inferred and their estimated frequencies. Effects of *CYP4F2*, *CYP4F11*, and *CYP4F12* haplotypes on hepatic mRNA expression of the *CYP4F* gene cluster were evaluated and significant associations are reported in **Table 3**.

**Table 3:** Genotype–phenotype correlation of *CYP4F2*, *CYP4F11*, and *CYP4F12* haplotypes and hepatic mRNA expression.

Gene				mRNA expression							
				*CYP4F2*			*CYP4F11*			*CYP4F12*		
	Haplotype identification code (see **Figure 3**)	SNP code	Haplotype sequence	*P*-value	↑ or ↓	*R*^2^ (%)	*P*-value	↑ or ↓	*R*^2^ (%)	*P*-value	↑ or ↓	*R*^2^ (%)
*CYP4F12*	1A	1112	AGTG	0.594	–	–	0.864	–	–	**4.13 × 10^-4^**	**↓**	**14.4**
	1C	2121	GGAA	0.894	–	–	0.468	–	–	**4.11 × 10^-5^**	**↑**	**19.2**
*CYP4F12*	2C	121	AGT	0.940	–	–	0.804	–	–	**2.82 × 10^-5^**	**↑**	**17.9**
	2D	212	GAC	0.759	–	–	0.912	–	–	**5.60 × 10^-3^**	**↓**	**10.3**
Intergenic	3D	1121	AGTT	0.332	–	–	0.485	–	–	**2.69 × 10^-4^**	**↑**	**15.2**
*CYP4F2*	4A	21	AT	**0.030**	**↑**	**7.9**	0.313	–	–	**0.030**	**↓**	**8.3**
*CYP4F2*	5A	121112111	TGCGGTGGG	**1.71 × 10^-3^**	**↑**	**12.6**	**5.96 × 10^-4^**	**↓**	**13.8**	0.595	–	–
*CYP4F2*	6B	22	AA	0.653	–	–	**0.033**	**↓**	**5.8**	0.274	–	–
	6C	21	AG	0.352	–	–	0.138	–	–	0.864	–	–
*CYP4F2*	7D	11211	CGGCC	0.496	–	–	0.095	–	–	0.604	–	–
*CYP4F11*	8B	112	TGC	**0.029**	**↓**	**7.0**	0.350	–	–	0.629	–	–
	8D	121	TAT	**0.027**	**↑**	**8.8**	0.054	–	–	0.997	–	–
*CYP4F11*	9B	22	AG	0.829	–	–	0.865	–	–	0.867	–	–
	9C	21	AT	**0.026**	**↑**	**9.4**	0.216	–	–	0.385	–	–

*Statistically significant haplotypes with a P < 0.05 are shown in bold. R^2^ refers to coefficient of determination. Haplotype identification code refers to the SNP constitution given in **Figure 3**. SNPs are coded as 1 or 2 (1 = frequent allele, 2 = minor allele).*

**FIGURE 4:**
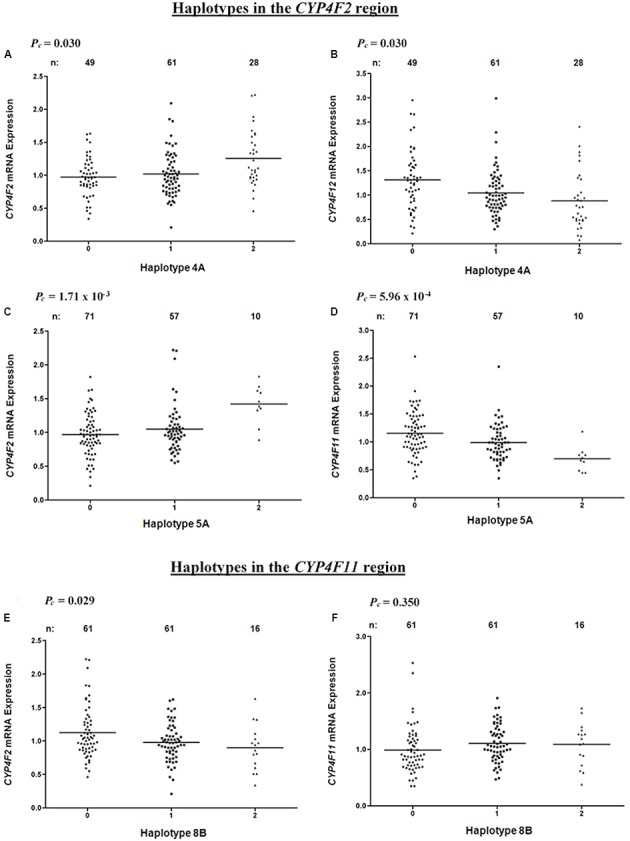
Levels of hepatic *CYP4F2*, *CYP4F11*, *CYP4F12* mRNA in normal liver tissue donated from 149 patients in relation to haplotypes across the *CYP4F2*-*CYP4F11* locus. **(A,B)** Haplotype 4A harboring sequence ‘AT’; **(C,D)** Haplotype 5A harboring sequence ‘TGCGGTGGG’; **(E,F)** Haplotype 8B harboring sequence ‘TGC.’ *FDR*-corrected *P*-values (*P_c_*) are shown in the upper left corner. Each dot represents an individual and the solid lines represent the mean values.

Haplotype 4A harboring the sequence ‘AT’ with a frequency of 43.3% was associated with a significant increase in hepatic *CYP4F2* (*P_c_* = 0.030, *R*^2^ = 7.9%; **Figure 4A**) and reduced *CYP4F12* (*P_c_* = 0.030, *R*^2^ = 8.3%; **Figure 4B**) mRNA expression, mirroring the effect of rs2189784. Corresponding to the effect of rs2108622, haplotype 5A ‘TGCGGTGGG’ (frequency = 28.3%) was significantly associated with increased *CYP4F2* (*P_c_* = 1.71 × 10^-3^, *R*^2^ = 12.6%; **Figure 4C**) and decreased *CYP4F11* (*P_c_* = 5.96 × 10^-4^, *R*^2^ = 13.8%; **Figure 4D**) mRNA expression. Resembling the effect of rs1060467, haplotype 8B (sequence ‘TGC,’ frequency = 33.2%) was associated with down-regulation of *CYP4F2* (*P_c_* = 0.029, *R*^2^ = 7.0%; **Figure 4E**) and showed a non-significant up-regulating effect on *CYP4F11* (*P_c_* = 0.350; **Figure 4F**) mRNA expression.

### Competing Effects of *CYP4F11* rs1060467 and *CYP4F2* rs2108622 on Warfarin Stable Dose

**FIGURE 5:**
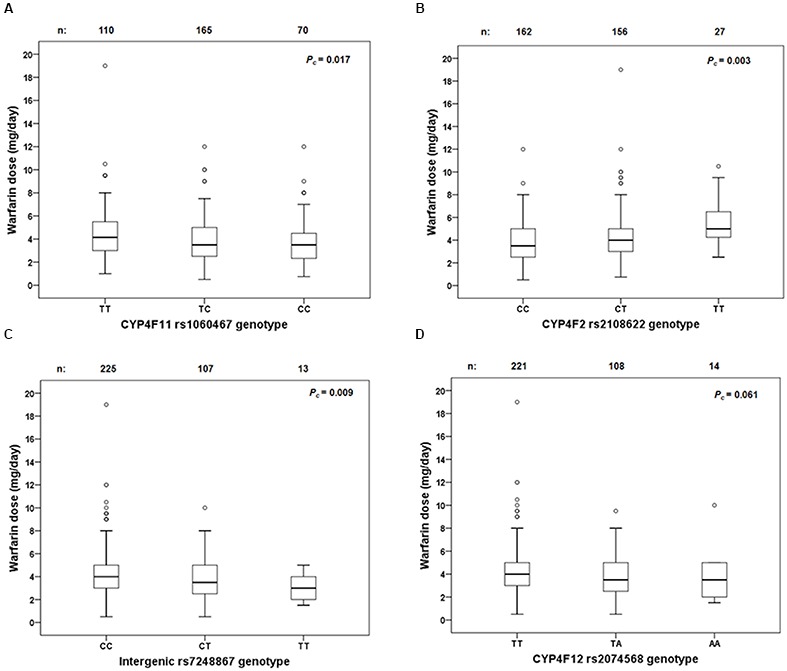
Box and whisker plots showing the distribution of stable warfarin daily doses based on genotype groups in **(A)** *CYP4F11* rs1060467; **(B)** *CYP4F2* rs2108622; **(C)** intergenic rs7248867; **(D)** *CYP4F12* rs2074568. Boxes represent 25th–75th percentiles of warfarin doses, whiskers represent 5th–95th percentiles, and solid lines represent median dose in each group. Open dots represent outliers. FDR-corrected *P*-values (*P_c_*) are shown on the upper right corner. Out of the 711 patients recruited prospectively, 345 achieved warfarin stable dose.

Linkage disequilibrium analysis of genotypes in our 149 livers revealed that *CYP4F11* rs1060467 and *CYP4F2* rs2108622 were moderately correlated with LD estimates of *r*^2^/D′ = 0.21/1.00. To assess the roles of rs1060467 and rs2108622 in warfarin response, we tested their association with the clinical outcome of warfarin stable dose in our prospective cohort of warfarin-treated patients (*n* = 711). Demographics of the 711 patients are summarized in **Table 4**. Among the 711 patients investigated, 345 achieved warfarin stable dose. **Figure 5A** illustrates warfarin stable dose established in patients, stratified by *CYP4F11* rs1060467 genotype. Patients with a C allele exhibited reduced stable dose requirements (mg/day: TT = 4.6 ± 0.2, TC = 3.9 ± 0.1, CC = 3.8 ± 0.2; *P_c_* = 0.017). The proportion of warfarin dose variability explained by rs1060467 was 2.6%. Conversely, as depicted in **Figure 5B**, patients carrying the *CYP4F2* rs2108622 T allele showed increased warfarin stable dose requirements (mg/day: CC = 3.7 ± 0.1, CT = 4.3 ± 0.2, TT = 5.3 ± 0.4; *P_c_* = 0.003) and rs2108622 accounted for 4.3% of warfarin dose variance.

**Table 4:** Clinical profile of 711 warfarin patients.

Characteristic	*N* (%)
Gender: male	394 (55.4)
Age^a^ in years, mean (range)	69 (19-95)
BMI^b^, mean (range)	28 (13-55)
**Ethnicity**	
White	710 (99.9)
Black-Caribbean	1 (0.1)
**Indication for warfarin**	
Atrial fibrillation	469 (66.0)
Pulmonary embolism	110 (15.5)
Deep vein thrombosis	74 (10.4)
Cerebrovascular accident and transient ischemic attacks	44 (6.2)
Mechanical heart valve replacement	8 (1.1)
Myocardial infarction	4 (0.6)
Dilated left atrium	2 (0.3)
Other^c^	36 (5.1)
**Co-morbidity**	
Cardiovascular disease	574 (80.7)
Musculoskeletal problems	426 (59.9)
Respiratory disease	268 (37.7)
Gastrointestinal disease	253 (35.6)
Neurological disease	156 (21.9)
Urological condition	132 (18.6)
Renal disease	75 (10.5)
History of falls	58 (8.2)
Hepatic disease	34 (4.8)

*BMI, body mass index. ^a^Age missing for nine patients. ^b^BMI missing for six patients. ^c^Other indications include: prevention of clotting in arm for dialysis; systemic lupus erythematosus; anti-phospholipid syndrome; short saphenous vein thrombosis; valvular heart disease; saggital sinus thrombosis; dilated left ventrical; occluded graft in leg; pulmonary hypertension; apical aneurysm; uticaria with angiodema; femoral embolectomy; aortic and mitral regurgitation; ischemic colitis; mitral stenosis; and post-surgery.*

By segregating the patients according to their haplotypes for *CYP4F2* rs2108622 and *CYP4F11* rs1060467 as illustrated in **Table 5**, it can be seen that there were small dose changes in patients carrying haplotypes consisting of *CYP4F2* rs2108622 wild-type genotype and *CYP4F11* rs1060467 variant genotype and vice versa.

**Table 5:** Relationship between *CYP4F2* and *CYP4F11* SNPs and stable warfarin dose requirement^∗^.

Warfarin dose (mg/day)			
*CYP4F2* rs2108622 genotype		*CYP4F11* rs1060467 genotype	
		TT (n)	CT and CC (n)
CC (n)	3.7 (162)	4.0 (18)	3.7 (144)
CT and TT (n)	4.4 (183)	4.7 (92)	4.0 (91)

*^∗^Out of 711 patients recruited prospectively, 345 achieved warfarin stable dose.*

### Imputation and Conditional Analysis

To explore the presence of additional signals at the *CYP4F* loci, genotype imputations were carried out across the 380 kb genomic region encompassing the *CYP4F12-CYP4F2-CYP4F11* region. Although additional SNPs showed significant associations with warfarin stable dose, the associations with *CYP4F2* rs2108622 and *CYP4F11* rs1060467 remained the most significant among all the *CYP4F2* and *CYP4F11* SNPs, respectively.

We also performed conditional analyses to evaluate the independence of association between *CYP4F2* rs2108622 and *CYP4F11* rs1060467. When we conditioned on *CYP4F11* rs1060467, a reduction in both magnitude and significance was seen with the association of warfarin stable dose with *CYP4F2* rs2108622 (β_initial_ = 0.078, β_conditional_ = 0.063, *P*_c_initial__ = 0.003, *P*_c_conditional__ = 0.05). When we conditioned on *CYP4F2* rs2108622, the association of warfarin stable dose with *CYP4F11* rs1060467 disappeared (*P*_c_initial__ = 0.017, *P*_c_conditional__ = 0.418). These results suggest that *CYP4F2* rs2108622 can explain the association signal for *CYP4F11* rs1060467 or vice versa.

### Warfarin Dose Prediction Algorithms

To assess whether the inclusion of *CYP4F2* rs2108622 and/or *CYP4F11* rs1060467 improves warfarin dose predictive accuracy, we developed a clinical algorithm and several pharmacogenetic algorithms as shown in **Table 6**. The clinical algorithm included four predictors which were found significant in the univariate analyses: age, BMI, gender and baseline clotting factor II level, which explained 15.7% of warfarin dose variability. The pharmacogenetic algorithm included *CYP2C9^∗^2* rs1799853, *CYP2C9^∗^3* rs1507910, and *VKORC1* rs9923231 genotypes in addition to the clinical factors and accounted for a 32.3% increase in warfarin dose variability, with a marked improvement in dose prediction accuracy. The addition of *CYP4F2* rs2108622 or *CYP4F11* rs1060467 to the pharmacogenetic algorithm explained a further 0.5–0.7% in warfarin dose variability with a modest increase in prediction accuracy (∼1% decrease in MAE and 1.2% increase in the number of predicted dose which fell within ±20% of the observed warfarin dose). When both rs2108622 and rs1060467 were incorporated into the pharmacogenetic algorithm, there was a modest increase in the number of predicted doses which fell within ±20% of the observed warfarin dose (∼2%).

**Table 6:** Comparison of predicted stable warfarin doses to actual stable warfarin doses using different prediction algorithms.

Prediction algorithm^∗^	Variables included	MAE ± SE (mg/week)	*R*^2^ Adj (%)	Within ± 20% of observed dose (%)
Clinical	Age, BMI, Gender, Baseline Factor II	9.08 ± 1.06	15.7	43.7
Clinical + *CYP2C9^∗^2* + *CYP2C9^∗^3* + *VKORC1*	Age, BMI, Gender, Baseline Factor II, rs1799853, rs1057910, rs9923231	7.32 ± 0.88	48.0	47.3
Clinical + *CYP2C9^∗^2* + *CYP2C9^∗^3* + *VKORC1* + *CYP4F2*	Age, BMI, Gender, Baseline Factor II, rs1799853, rs1057910, rs9923231, rs2108622	7.26 ± 0.88	48.5	48.5
Clinical + *CYP2C9^∗^2* + *CYP2C9^∗^3* + *VKORC1* + *CYP4F11*	Age, BMI, Gender, Baseline Factor II, rs1799853, rs1057910, rs9923231, rs1060467	7.24 ± 0.88	48.7	48.5
Clinical + *CYP2C9^∗^2* + *CYP2C9^∗^3* + *VKORC1* + *CYP4F2* + *CYP4F11*	Age, BMI, Gender, Baseline Factor II, rs1799853, rs1057910, rs9923231, rs2108622, rs1060467	7.22 ± 0.88	48.6	49.4

*^∗^Of the 345 patients who achieved stable warfarin dose, data on age, BMI and CYP2C9^∗^2 rs1799853 were missing for 4, 3, and 2 individuals, respectively. Therefore only 336 patients were included in the prediction algorithms above. BMI, body mass index; MAE, mean absolute error; SE, standard error; R^2^ Adj, adjusted coefficient of determination.*

### *In Silico* Genotype–Phenotype Analysis

To explore whether the SNP-gene effects observed in our cohort of human livers was also present in other published studies, we assessed eQTLs in the region encompassing *CYP4F2*, *CYP4F11*, and *CYP4F12* genes using the publicly available eQTL database hosted by the Pritchard laboratories at the University of Chicago. **Table 7** outlines the significant SNP-gene associations available on the eQTL database. Of particular interest is the positive association of rs7248867, a SNP located between *CYP4F12* and *CYP4F2*, with *CYP4F11* transcript levels in livers from individuals of European descent. Using genotype data available on HapMap, LD analysis revealed that this intergenic SNP is in moderate LD with both rs2189784 (*D*′ = 1.0, *r*^2^ = 0.103) and rs2108622 (*D*′ = 1.0, *r*^2^ = 0.046). rs7248867 also tags several SNPs (using *r*^2^ > 0.8) including a *CYP4F12* intronic SNP, rs2074568 (*D*′ = 1.0, *r*^2^ = 0.837) which was analyzed in our cohort of 149 individuals who had donated liver samples. rs2074568 showed a significant association with increased hepatic *CYP4F12* mRNA expression (*P_c_* = 1.49 × 10^-5^) but not with *CYP4F11* (*P* = 0.25) and *CYP4F2* (*P* = 0.537).

**Table 7:** Expression quantitative trait loci (eQTLs) in the *CYP4F12-CYP4F2-CYP4F11* gene cluster region.

SNP	SNP localization	SNP chromosomal location	Target eQTL gene	*P*-value	Tissue	Study
rs7246556	5′ upstream of *CYP4F12*	15637511	*SLC35E1*	9.75439E-05	Monocytes	[52]
rs4808351	5′ upstream of *CYP4F12*	15638714	*SLC35E1*	6.21012E-05	Monocytes	[52]
rs4807967	5′ upstream of *CYP4F12*	15638931	*SLC35E1*	6.21012E-05	Monocytes	[52]
rs4808352	5′ upstream of *CYP4F12*	15638996	*SLC35E1*	0.00011855	Monocytes	[52]
rs10409673	5′ upstream of *CYP4F12*	15640453	*SLC35E1*	6.21012E-05	Monocytes	[52]
rs7251084	5′ upstream of *CYP4F12*	15641041	*SLC35E1*	6.21012E-05	Monocytes	[52]
rs7259028	5′ upstream of *CYP4F12*	15641245	*SLC35E1*	6.21012E-05	Monocytes	[52]
rs7248867	Intergenic, between CYP4F12 and CYP4F2	15731204	*CYP4F11*	8.23E-05	Liver	[49]
rs2074901	*CYP4F2*	15858422	*BRD4*	1.80053E-05	Monocytes	[52]
rs2074902	*CYP4F2*	15869099	*BRD4*	1.80053E-05	Monocytes	[52]
rs1060463	*CYP4F11*	15886176	*ILVBL*	9.96552E-05	Monocytes	[52]
rs6512075	*CYP4F11*	15899334	*ILVBL*	9.96552E-05	Monocytes	[52]
rs3746154	*CYP4F11*	15899390	*ILVBL*	9.96552E-05	Monocytes	[52]
rs3746156	*CYP4F11*	15896494	*ILVBL*	9.96552E-05	Monocytes	[52]
rs2219358	*CYP4F11*	15896517	*ILVBL*	9.96552E-05	Monocytes	[52]
rs2305803	*CYP4F11*	15888067	*ILVBL*	9.96552E-05	Monocytes	[52]
rs17641483	5′ upstream of *CYP4F11*	15919371	*CYP4F11*	6.40767E-13	Monocytes	[52]

*Chromosomal positions are given in base pairs from the p-telomere of chromosome 19, as per HapMap Data release 27, NCBI B36 assembly, dbSNP b126. BRD4, bromodomain containing protein 4; ILVBL, acetolactate synthase-like protein; SLC35E1, solute carrier family 35, member E1.*

### Effect of Intergenic rs7248867 and *CYP4F12* rs2074568 on Warfarin Stable Dose

Genotypes from the 1000 genomes project were imputed to evaluate the effect of rs7248867 and rs2074568 on warfarin stable dose. As illustrated in **Figure 5C**, patients carrying the minor rs7248867 T-allele required lower warfarin doses compared to patients carrying the major C-allele (mg/day: CC = 4.3 ± 0.1, CT = 3.7 ± 0.2, TT = 3.2 ± 0.4; *P_c_* = 0.009). The association of rs2074568 was not significant after FDR but showed a recessive effect on warfarin dose requirements (**Figure 5D**) with the minor A-allele (mg/day: TT = 4.3 ± 0.1, TA = 3.7 ± 0.2, AA = 3.7 ± 0.6; *P_c_* = 0.061).

To assess the independence of these two SNPs to *CYP4F2* rs2108622, conditional analyses were performed. When conditioned on rs7248867, the association of *CYP4F2* rs2108622 with warfarin stable dose decreased in both magnitude and significance (β_initial_ = 0.078, β_conditional_ = 0.065, *P*_c_initial__ = 0.003, *P*_c_conditional__ = 0.015). When we conditioned on rs2074568, a reduction in magnitude and significance were also observed with *CYP4F2* rs2108622 (β_conditional_ = 0.069, *P*_c_conditional__ = 0.009). These results suggest that rs7248867 and rs2074568 are correlated with *CYP4F2* rs2108622.

## Discussion

To elucidate whether the association between genotype and gene expression reflected *cis*-acting regulatory effects on the *CYP4F* gene cluster, we conducted a comprehensive investigation looking at the effects of *CYP4F2*, *CYP4F11*, and *CYP4F12* polymorphisms on the hepatic expression levels of *CYP4F2, CYP4F8, CYP4F11*, and *CYP4F12* mRNA in a Caucasian population. We report for the first time that SNPs and extended haplotypes in *CYP4F2*, *CYP4F11*, and *CYP4F12* affect the mRNA expression levels of *CYP4F2, CYP4F11*, and *CYP4F12* in human liver tissues and that *CYP4F11* plays a role in warfarin response.

Unlike McDonald et al. (2009), our study observed a significant association between the *CYP4F2* rs2108622 SNP and an increase in *CYP4F2* mRNA expression, explaining over 12% of the variability in *CYP4F2* mRNA expression. This may reflect our larger sample size (*n* = 149) of livers compared with the previous study (McDonald et al., 2009). Consistent with the fact that the *CYP4F* genes are highly homologous and show extensive LD, our data show that SNPs in one *CYP4F* gene can have an effect on the expression of another *CYP4F* gene. In fact, rs2108622 in *CYP4F2* was associated with decreased *CYP4F11* mRNA expression accounting for nearly 14% of *CYP4F11* hepatic mRNA expression. Comparatively, the haplotype harboring this *CYP4F2* variant also displayed similar associations. Conversely, a variant in the 3′ UTR of the *CYP4F11* region, rs1060467, was associated with decreased *CYP4F2* mRNA expression, accounting for 7% of the variability in *CYP4F2* mRNA expression. The *CYP4F11* haplotype comprising the minor rs1060467 C-allele also had a corresponding recessive effect on *CYP4F2* mRNA expression.

Given these mutual genotype–phenotype relationships and the fact that both CYP4F2 and CYP4F11 had been identified as equally efficient vitamin K ω-hydroxylases (Edson et al., 2013), we hypothesized that rs1060467 may play a role in warfarin stable dose. Using our GWAS data previously conducted in 711 prospective patients on warfarin therapy, of which 345 patients achieved warfarin stable dose, rs1060467 explained 2.6% of warfarin dose variability, while rs2108622 accounted for 4.3%, similar to previous reports (Caldwell et al., 2008; Borgiani et al., 2009; Perez-Andreu et al., 2009). Interestingly, the association of rs1060467 with warfarin dose was opposite to that seen with rs2108622, confirming the compensatory effects *CYP4F2* and *CYP4F11* polymorphisms have on hepatic *CYP4F2* mRNA. However, when conditional analyses were performed using SNP rs2108622, the magnitude and significance level for rs1060467 were substantially attenuated, suggesting that rs1060467 and rs2108622 are dependent loci and are both likely to contribute to the same signal at the *CYP4F2-CYP4F11* region. Indeed, our pharmacogenetic algorithms incorporating *CYP4F11* rs1060467 or *CYP4F2* rs2108622 or both *CYP4F11* rs1060467 and *CYP4F2* rs2108622, explained a similar increase in warfarin dose variability with modest improvement in prediction accuracy (1–2%), indicating that just one of these SNPs can explain the effect on warfarin dose variability. The opposing effects between *CYP4F11* rs1060467 and *CYP4F2* rs2108622 in the *CYP4F* gene cluster do not appear to affect warfarin dose requirement.

Our present study also showed a significant association of rs2189784, a SNP located 30 kb downstream of *CYP4F2*, with differences in mRNA expression of *CYP4F2* and *CYP4F12*. Interestingly, we have previously reported this SNP to play a role in time taken to achieve therapeutic INR in patients on prospective warfarin therapy (Zhang et al., 2009). Likewise, the haplotype containing the minor A-allele of variant rs2189784 (haplotype 4A) was also significantly associated with increasing *CYP4F2* and decreasing *CYP4F12* mRNA expression. These results suggest that the previously observed association between rs2189784 and time to therapeutic INR (Zhang et al., 2009) may be mediated through an effect on *CYP4F2* and *CYP4F12* mRNA and SNPs in *CYP4F12* may affect *CYP4F2* mRNA expression. Evaluation of variants across the *CYP4F12* region however, did not show any SNPs to be associated with *CYP4F2* mRNA expression.

*In silico* eQTL analysis provided further insights into the complexity of the regulation of the *CYP4F* gene cluster. *CYP4F11* mRNA expression was associated with an intergenic SNP between *CYP4F12* and *CYP4F2*, rs7248867. This SNP is tagged by a *CYP4F12* intronic SNP (rs2074568) genotyped in our study. These two SNPs were however, not present on the GWAS platform. Imputations were therefore performed and a trend for reduced warfarin stable dose was seen with these two SNPs. However, our conditional analyses suggest that the association signals found with rs7248867 and rs2074568 could be explained by *CYP4F2* rs2108622.

A limitation of our study is that we did not investigate protein expression levels of the different CYP4F isoforms. The reason for this is that the protein sequences of CYP4F2, CYP4F11 and CYP4F12 share 81–93% similarity (Hirani et al., 2008) and currently available antibodies are likely to exhibit high level of cross-reactivity, decreasing the specificity of protein detection. New technologies such as gene editing could be employed to evaluate the function of these *CYP4F* genes.

## Conclusion

We have effectively examined sequence variations across the three *CYP4F* genes – *CYP4F2*, *CYP4F11*, and *CYP4F12* and their effect on mRNA expression. From a clinical perspective, our data show the complexity of gene–gene interactions, where competing effects of different SNPs within the same gene cluster can cancel out the level of *CYP4F2* mRNA and warfarin daily doses required to maintain anticoagulation. As a result, the overall effect of SNPs in *CYP4F2* and *CYP4F11* on warfarin dose variability is very small in our population. However, in other populations with different linkage patterns the influence of *CYP4F* SNPs may be larger. It is possible that additional variants which are rare and functionally active may be important other than the SNPs genotyped in our study, and resequencing of the *CYP4F2*, *CYP4F11* and *CYP4F12* genes in appropriately phenotyped patients on warfarin may help identify these.

## Author Contributions

MP, UZ, and PD designed the research study; JZ, KK, and SB performed the experiments; JZ, KK, AJ, BF, AA, SB, and UZ analyzed the results; JZ, KK, AA, UZ, and MP wrote the manuscript; all authors read and approved the final manuscript.

## Conflict of Interest Statement

The authors declare that the research was conducted in the absence of any commercial or financial relationships that could be construed as a potential conflict of interest.

## Footnotes

1 https://www.mysequenom.com/Tools

2 http://cran.r-project.org/web/packages/genetics/index.html

3 http://eqtl.uchicago.edu/cgi-bin/gbrowse/eqtl/
