# Supplementary material for: Effect of Genetic Variability in the CYP4F2, CYP4F11, and CYP4F12 Genes on Liver mRNA Levels and Warfarin Response
Source: Front Pharmacol. 2017 May 31;8:323. doi: 10.3389/fphar.2017.00323 (PMC5449482; doi:10.3389/fphar.2017.00323)
Supplement: Supplementary file 4 [file Data_Sheet_1.PDF]

Supplementary Results

Interaction between CYP4F2, CYP4F11 and CYP4F12

We used MetaCore™ to put *CYP4F2*, *CYP4F11* and *CYP4F12* into a cellular context to evaluate the significance of gene networks that these three genes participate in and to identify regulatory cascades that lead to or from these genes. Supplementary Figure 3 highlights the network interactions of nuclear transcriptional factors relating to the gene expression of *CYP4F2*, *CYP4F11* and *CYP4F12*. Several nuclear transcription factors including pregnane X receptor (PXR), aryl hydrocarbon receptor (AHR), activator protein 1(AP-1), peroxisome proliferator-activated receptor (PPAR) alpha, sterol regulatory element-binding protein 1 (SREBP-1), retinoid X receptor (RXR) and retinoic acid receptor (RAR) are known to be involved in the metabolism and clearance of diverse endogenous and exogenous compounds as well as gene activation (Pavek and Dvorak 2008; Zhou et al., 2009).

Supplementary Discussion

Interaction between CYP4F2, CYP4F11 and CYP4F12

Our network analysis shows that the regulation of *CYP4F2*, *CYP4F11* and *CYP4F12* expression is co-ordinated via numerous nuclear transcription factors including AP-1, RXR, RAR and SREBP, which are also known to be involved in the regulation of a number of other P450 isoforms (Rushmore and Kong 2002; Roth et al., 2008). This is consistent with data from various cell lines: AP-1 and RXR have been shown to regulate *CYP4F11* expression (Wang et al., 2010) in human keratinocyte-derived HaCaT cells; RXR stimulated whilst RAR repressed *CYP4F2* expression in the HepG2 cell line (Zhang et al., 2000; Zhang and Hardwick 2000); SREBP mediated the induction of *CYP4F2* expression by statins in primary human hepatocytes and HepG2 cells (Hsu et al., 2007); and PXR has been shown to regulate *CYP4F2* expression in healthy human lymphocytes (Siest et al., 2008) and *CYP4F12* expression in primary human hepatocytes (Hariparsad et al., 2009). Further work is required to further assess the cross-regulation of the *CYP4F* genes.

References

Hariparsad, N., Chu, X., Yabut, J., Labhart, P., Hartley, D.P., Dai, X., and Evers, R. (2009). Identification of pregnane-X receptor target genes and coactivator and corepressor binding to promoter elements in human hepatocytes. *Nucleic acids research* 37**,** 1160-1173.

Hsu, M.H., Savas, U., Griffin, K.J., and Johnson, E.F. (2007). Regulation of human cytochrome P450 4F2 expression by sterol regulatory element-binding protein and lovastatin. *J Biol Chem* 282**,** 5225-5236.

Pavek, P., and Dvorak, Z. (2008). Xenobiotic-induced transcriptional regulation of xenobiotic metabolizing enzymes of the cytochrome P450 superfamily in human extrahepatic tissues. *Current drug metabolism* 9**,** 129-143.

Roth, A., Looser, R., Kaufmann, M., and Meyer, U.A. (2008). Sterol regulatory element binding protein 1 interacts with pregnane X receptor and constitutive androstane receptor and represses their target genes. *Pharmacogenetics and genomics* 18**,** 325-337.

Rushmore, T.H., and Kong, A.N.T. (2002). Pharmacogenomics, regulation and signaling pathways of phase I and II drug metabolizing enzymes. *Current drug metabolism* 3**,** 481-490.

Siest, G., Jeannesson, E., Marteau, J.-B., Samara, A., Marie, B., Pfister, M., and Visvikis-Siest, S. (2008). Transcription Factor and Drug-Metabolizing Enzyme Gene Expression in Lymphocytes from Healthy Human Subjects. *Drug Metabolism and Disposition* 36**,** 182-189.

Wang, Y., Bell, J.C., Keeney, D.S., and Strobel, H.W. (2010). Gene regulation of CYP4F11 in human keratinocyte HaCaT cells. *Drug metabolism and disposition: the biological fate of chemicals* 38**,** 100-107.

Zhang, X., Chen, L., and Hardwick, J.P. (2000). Promoter activity and regulation of the CYP4F2 leukotriene B(4) omega-hydroxylase gene by peroxisomal proliferators and retinoic acid in HepG2 cells. *Archives of biochemistry and biophysics* 378**,** 364-376.

Zhang, X., and Hardwick, J.P. (2000). Regulation of CYP4F2 leukotriene B4 omega-hydroxylase by retinoic acids in HepG2 cells. *Biochemical and biophysical research communications* 279**,** 864-871.

Zhou, C., Verma, S., and Blumberg, B. (2009). The steroid and xenobiotic receptor (SXR), beyond xenobiotic metabolism. *Nuclear receptor signaling* 7**,** e001.
