# Supplementary material for: Effect of Genetic Variability in the CYP4F2, CYP4F11, and CYP4F12 Genes on Liver mRNA Levels and Warfarin Response
Source: Front Pharmacol. 2017 May 31;8:323. doi: 10.3389/fphar.2017.00323 (PMC5449482; doi:10.3389/fphar.2017.00323)
Supplement: Supplementary file 5 [file Image_1.PDF]

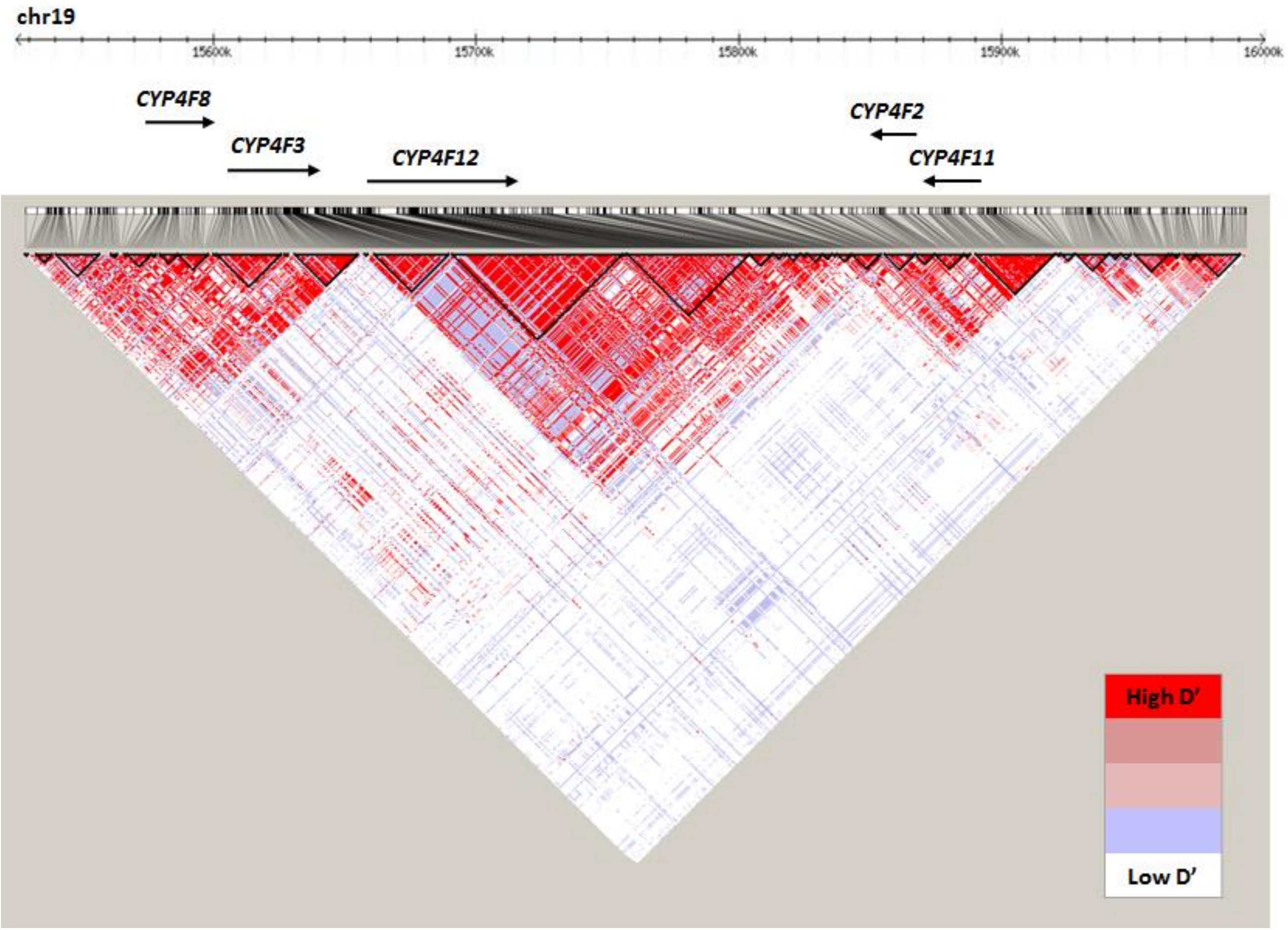

Supplementary Figure 1. Genomic structure of the *CYP4F* gene cluster encompassing *CYP4F2, CYP4F3, CYP4F8, CYP4F11* and *CYP4F12*. LD pattern and haplotype blocks were generated using HaploView version 4.2 based on the CEU population genotype data on chromosome 19p13 region 15529000 – 16040000, obtained from HapMap data release 27, NCBI build 36 assembly.
