# Supplementary figures and images for: Effect of Genetic Variability in the CYP4F2, CYP4F11, and CYP4F12 Genes on Liver mRNA Levels and Warfarin Response
Source: Front Pharmacol. 2017 May 31;8:323. doi: 10.3389/fphar.2017.00323 (PMC5449482; doi:10.3389/fphar.2017.00323)

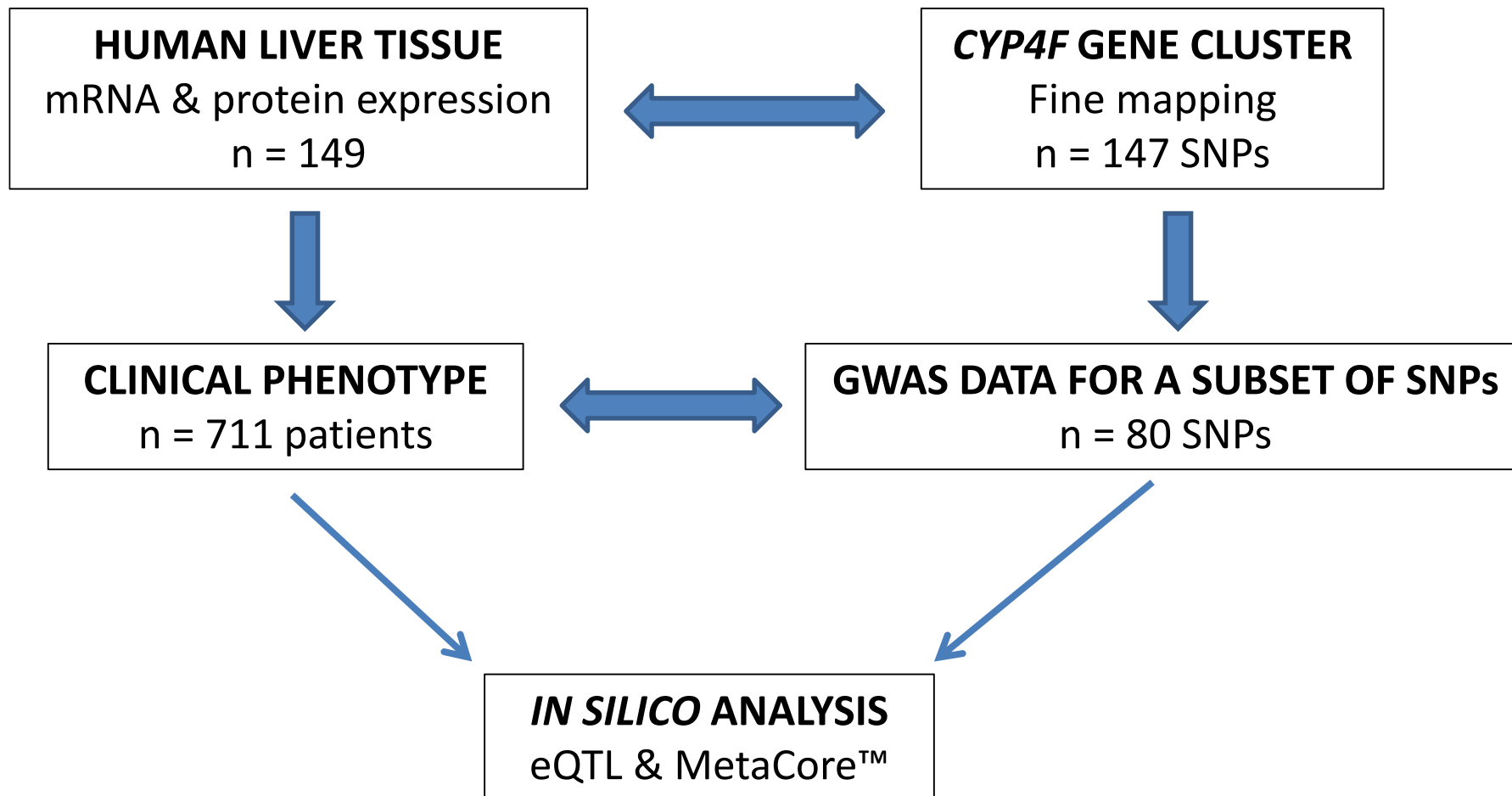

Supplementary Figure 2. Genotype-phenotype correlation

Supplement: Supplementary file 6 [file Image_2.PDF]
