# Supplementary material for: Effect of Genetic Variability in the CYP4F2, CYP4F11, and CYP4F12 Genes on Liver mRNA Levels and Warfarin Response
Source: Front Pharmacol. 2017 May 31;8:323. doi: 10.3389/fphar.2017.00323 (PMC5449482; doi:10.3389/fphar.2017.00323)
Supplement: Supplementary file 7 [file Image_3.PDF]

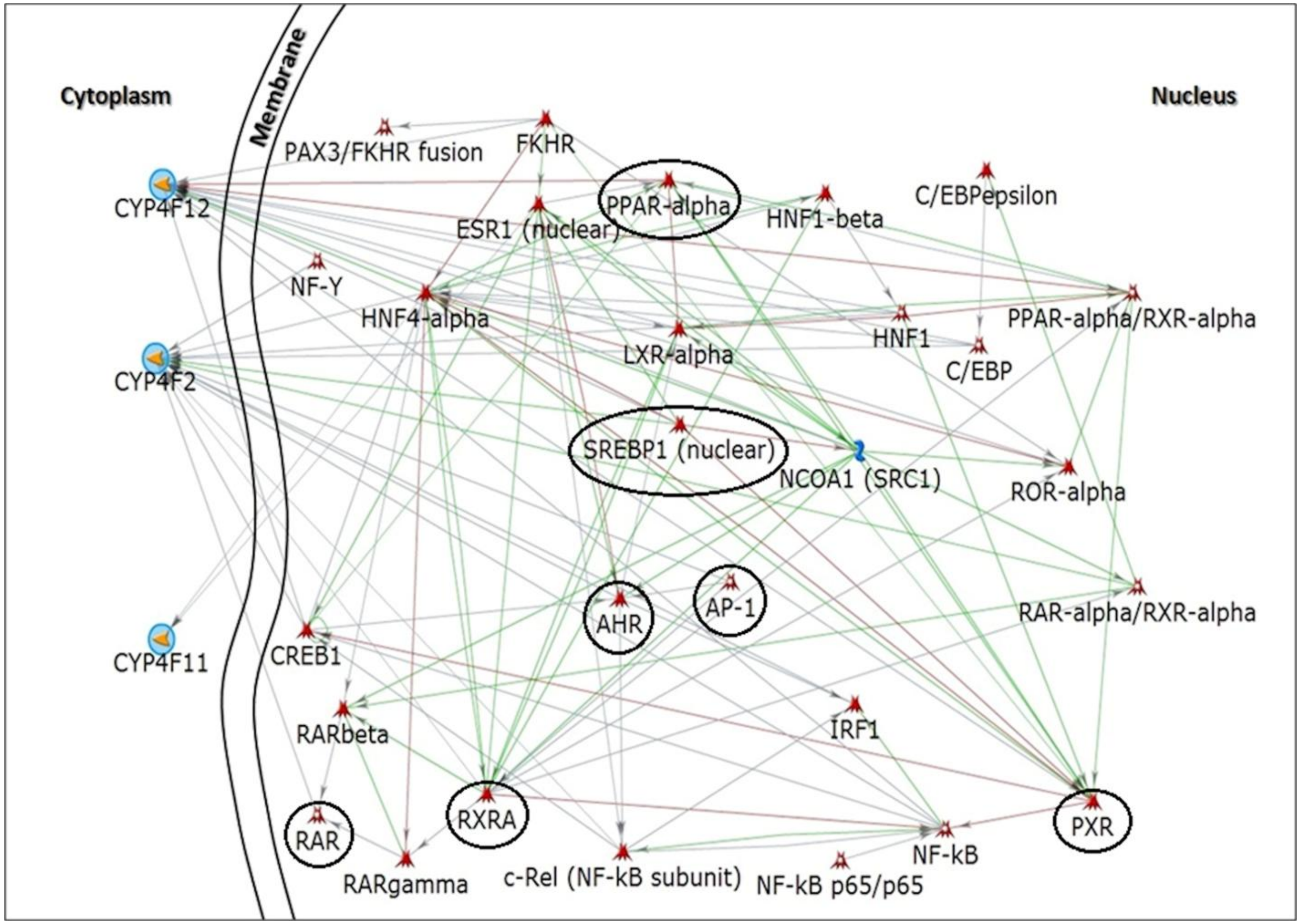

Supplementary Figure 3. GeneGo graphic illustrating the interaction of nuclear factors with *CYP4F2*, *CYP4F11* and *CYP4F12* genes. Each connection represents a direct, experimentally confirmed, physical interaction between the objects.
